# Multi-Dimensional Validation of the Integration of Syntactic and Semantic Distance Measures for Clustering Fibromyalgia Patients in the Rheumatic Monitor Big Data Study

**DOI:** 10.3390/bioengineering11010097

**Published:** 2024-01-19

**Authors:** Ayelet Goldstein, Yuval Shahar, Michal Weisman Raymond, Hagit Peleg, Eldad Ben-Chetrit, Arie Ben-Yehuda, Erez Shalom, Chen Goldstein, Shmuel Shay Shiloh, Galit Almoznino

**Affiliations:** 1 Computer Science Department, Hadassah Academic College, Jerusalem 9101001, Israel; ayeletgo@hac.ac.il; 2 Medical Informatics Research Center, Department of Software and Information Systems Engineering, Ben Gurion University of the Negev, Beer Sheva 8410501, Israel; yshahar@bgu.ac.il (Y.S.);; 3 Rheumatology Unit, Hadassah Medical Center, Jerusalem 9112102, Israel; 4 Division of Internal Medicine, Hadassah Medical Center, Jerusalem 9112102, Israel; 5 Faculty of Dental Medicine, Hebrew University of Jerusalem, Israel; Big Biomedical Data Research Laboratory, Dean’s Office, Hadassah Medical Center, Jerusalem 91120, Israel; 6 Department of Oral Medicine, Sedation & Maxillofacial Imaging, Hadassah Medical Center, Faculty of Dental Medicine, Hebrew University of Jerusalem, Jerusalem 91120, Israel

**Keywords:** cluster analysis, machine learning algorithm, K-means, Big Data, fibromyalgia, rheumatic diseases

## Abstract

This study primarily aimed at developing a novel multi-dimensional methodology to discover and validate the optimal number of clusters. The secondary objective was to deploy it for the task of clustering fibromyalgia patients. We present a comprehensive methodology that includes the use of several different clustering algorithms, quality assessment using several syntactic distance measures (the Silhouette Index (SI), Calinski–Harabasz index (CHI), and Davies–Bouldin index (DBI)), stability assessment using the adjusted Rand index (ARI), and the validation of the internal semantic consistency of each clustering option via the performance of multiple clustering iterations after the repeated bagging of the data to select multiple partial data sets. Then, we perform a statistical analysis of the (clinical) semantics of the most stable clustering options using the full data set. Finally, the results are validated through a supervised machine learning (ML) model that classifies the patients back into the discovered clusters and is interpreted by calculating the Shapley additive explanations (SHAP) values of the model. Thus, we refer to our methodology as the clustering, distance measures and iterative statistical and semantic validation (CDI-SSV) methodology. We applied our method to the analysis of a comprehensive data set acquired from 1370 fibromyalgia patients. The results demonstrate that the K-means was highly robust in the syntactic and the internal consistent semantics analysis phases and was therefore followed by a semantic assessment to determine the optimal number of clusters (k), which suggested k = 3 as a more clinically meaningful solution, representing three distinct severity levels. the random forest model validated the results by classification into the discovered clusters with high accuracy (AUC: 0.994; accuracy: 0.946). SHAP analysis emphasized the clinical relevance of "functional problems" in distinguishing the most severe condition. In conclusion, the CDI-SSV methodology offers significant potential for improving the classification of complex patients. Our findings suggest a classification system for different profiles of fibromyalgia patients, which has the potential to improve clinical care, by providing clinical markers for the evidence-based personalized diagnosis, management, and prognosis of fibromyalgia patients.

## 1. Introduction

Fibromyalgia represents the most prevalent source associated with chronic wide-spread musculoskeletal pain, accompanied by fatigue and sleep disturbances, which are present for at least three months and not explained by any other medical condition [1]. Fibromyalgia patients may exhibit a variety of other somatic symptoms including functional impairment and psychiatric symptoms [2]. It is most common in women, and the prevalence rises with age [3,4,5]. The estimated prevalence is 6.4% (7.7% in women and 4.9% in men) in the United States [4], and 3.3 to 8.3% in Europe and South America [5]. The etiology and pathophysiology of fibromyalgia is currently not known, and there is no evidence of inflammation in the soft tissues [2]. It is considered a pain regulation disorder, often classified as central sensitization [6], due to alterations in central nervous system pain and sensory processing [7].

Identifying patient subgroups can assist in comprehending the modifiable risk factors associated with each cluster and optimize personalized therapeutic strategies [8]. This is important in fibromyalgia patients, as physicians may hesitate to accept them due to difficulty in controlling symptoms and a lack of information about treatments and causes [9]. Prior research identified subgroups of women with fibromyalgia based on various characteristics, such as pain, tender points, disability, sensory, cognitive, psychological, or physical features [10,11,12,13,14]. Previous clustering research on fibromyalgia excluded patients with trauma history, and comorbid systemic and rheumatological diseases [10]. However, it is important to include comorbidities and trauma since fibromyalgia is more frequent in rheumatic diseases [15]. Moreover, up to one-fourth of the patients had precipitating physical trauma [16], and psychological trauma; especially, childhood trauma is a risk factor for the fibromyalgia onset [17].

In recent years, machine learning (ML) has emerged as a pivotal tool in various fields, including the medical field [18,19] due to its ability to uncover patterns and insights from complex datasets. For instance, graph-based deep learning has been utilized for medical diagnosis [20], and inverse reinforcement learning (IRL) algorithms have optimized performance in complex systems [21]. These advancement in ML, particularly in clustering techniques, have shown promise in various medical applications [22,23,24]. The potential of ML in enhancing the understanding and treatment of complex medical conditions like fibromyalgia is significant, especially given the challenges in subgroup identification and the need for personalized treatment strategies.

Recent advances in clustering methods lack consensus on optimal methods and validation approaches. Therefore, the primary aim of our study is to address this unmet need by developing and evaluating a novel comprehensive multi-dimensional. clustering methodology. This methodology is designed to be broadly applicable in various contexts, with a specific emphasis on determining the optimal number of clusters in a given dataset. The secondary objective is the application of this developed methodology to the specific case of clustering fibromyalgia patients. This application is intended to demonstrate the utility of the methodology in a practical healthcare context, providing insights into the heterogeneity of fibromyalgia. By implementing the suggested novel clustering methodology, we aim to identify the optimal clustering approach for fibromyalgia patients and provide a generalizable method for other clinical datasets. This study presents a significant contribution to clustering methods and to clinical knowledge discovery, offering a robust and comprehensive novel clustering framework. Furthermore, unlike prior research in the fibromyalgia domain, which included dozens of [14] or several hundred patients [10,11,13], our study includes 1370 patients with a comprehensive documentation of their socio-demographics, comorbidities, symptoms, trauma, sleep, pain, functional problems, and treatment modalities. This enabled us to address the full heterogeneity of the population of fibromyalgia patients.

## 2. Methods

### 2.1. Data Source, Study Participants and Questionnaire

This research is part of the Rheumatic Monitor study, which focuses on advancing personalized medicine by identifying patterns that predict the severity of rheumatic diseases and treatment response [25]. In the Rheumatic Monitor study, we developed a mobile application for iPhone and Android operating systems that collects baseline and dynamic questionnaires and includes an option to report on pain attacks and visualize pain reports. More about the Rheumatic Monitor study and the application can be found on the research website: https://www.rheumaticmonitor.org/, accessed on 1 January 2024 [25].

We recruited 1370 fibromyalgia patients voluntarily from an Israeli fibromyalgia association who responded to a comprehensive questionnaire. In total, 163 features, 151 categorial and 12 numerical, were obtained via a the 28-question online survey, based on the Rheumatic Monitor application questionnaire, including variables for painful areas, co-morbidities, sleep problems, and other domains. The parameters used in the analyses are depicted in Figures 3–7.

**Eligibility criteria**

**Inclusion criteria:** Patients aged 18–99 years, with a fibromyalgia diagnosis given by their rheumatologist.

**Exclusion criteria:** Patients under 18 years, and pregnant women/breastfeeding women; patients under 18 years of age due to the need for additional ethical approvals required for minors and their distinct epidemiological and medical characteristics.

### 2.2. Ethical Approval

The study received approval from the Institutional Review Board (IRB) of Hadassah Medical Organization (HMO), approval number 0205-19-HMO. As the study only entailed anonymous survey analysis, an exemption from informed consent was granted by the IRB.

### 2.3. The Clustering, Distance Measures and Iterative Statistical and Semantic Validation (CDI-SSV) Methodology

**Figure 1:**
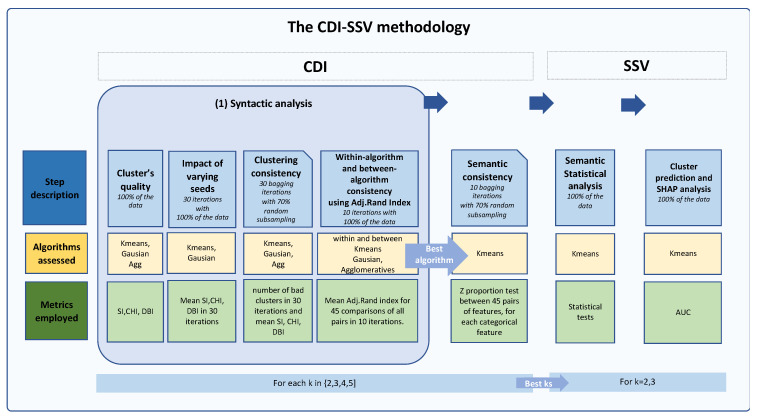
**The CDI-SSV Methodology**: An Integrated Approach for Clustering Validation. The figure provides an overview of the Clustering, Distance measures, and Iterative Statistical and Semantic Validation (CDI-SSV) methodology. The CDI phase serves as the initial step, involving the evaluation of cluster quality, the impact of different starting seeds, and the consistency of clusters across various algorithms and pre-defined values of k. Within- and between-consistency checks, along with evaluations of internal semantic consistency, are performed to assess the optimal algorithm and values of k. In the subsequent SSV phase, an external semantic analysis of the results is conducted, with a particular focus on the clinical context, thus enhancing the validation process. Finally, machine learning techniques are employed to validate the results, and their interpretation is facilitated by SHAP (Shapley additive explanations) values.

We propose a comprehensive multi-dimensional validation methodology for clustering fibromyalgia patients, integrating both syntactic (based on data’s quantitative attributes) and semantic (based on meaning) distance measures. Figure 1 illustrates this methodology. We refer to the first phase of our methodology as the **CDI** phase, a syntactic analysis that employs several **clustering** algorithms, and **distance** measures. These are followed by multiple **iterations** to evaluate the influence of varying initial seeds, clustering consistency with partial data, and within and between algorithm clustering consistency. Subsequently, the **SSV** phase utilizes **statistical** analysis to validate the clinical **semantics** of the potential clustering options that survived our rigorous pipeline. Finally, **validation** of the clusters is conducted using a supervised machine learning (ML) model to classify the patients back into the discovered clusters, and the interpretation is further enhanced through Shapley additive explanations (SHAP) analysis.

### 2.4. Data Scaling

Prior to clustering the dataset, we applied feature-wise scaling to the data using StandardScaler from sklearn.preprocessing, so each feature contributed equally to the analysis. This standardized each feature to a mean of zero and a standard deviation of one. Such standardization ensures that features with larger ranges do not disproportionately influence the clustering, thereby maintaining comparability across our dataset’s diverse features, such as clinical and demographic variables. We then applied various clustering algorithms available in the scikit-learn (sklearn) library in Python [26].

### 2.5. The CDI (Clustering, Distance Measures, and Iterative) Phase

#### 2.5.1. Clustering Algorithms

We evaluated and compared three widely-used clustering algorithms: K-means [27,28], Gaussian mixture [29,30], and agglomerative clustering [31], utilizing different linkage methods (complete, ward, average, and single) [32]). These algorithms were selected for their proven effectiveness in handling diverse data types and their widespread use in similar studies. For each of these algorithms, we employed the default parameters as implemented in the scikit-learn (sklearn) library in Python. This decision was made to ensure consistency with standard practices in the field and to facilitate reproducibility by other researchers. We also used Gower’s distance metric [33] as the distance function between data points, suitable for mixed data types like ours.

#### 2.5.2. Distance Measures

##### Syntactic Clustering-Quality Evaluation Metrics

To assess clustering quality, we used internal metrics like the silhouette index (SI) [34,35], Davies–Bouldin index (DBI) [36], and Calinski–Harabasz index (CHI) [37]. These metrics were chosen for their ability to provide a comprehensive assessment of clustering quality. The SI score provides insights into the matching of data points to their assigned clusters and neighboring clusters, with higher scores indicating better matching. The DBI score measures the separation between clusters, with lower scores indicating better separation. The CHI score indicates the degree of cluster definition, with higher scores representing better-defined clusters. For each algorithm and number k of clusters, we calculated the SI, DBI, and CHI. In addition to these metrics, we employed the adjusted Rand index (ARI) to quantify the similarity between two clustering solutions. The ARI score ranges from −1 to 1 (0: random correlation; 1: perfect correlation). These metrics collectively offer a balanced evaluation of cluster cohesion and separation, essential for our study’s objectives.

##### Assessment of the Clustering’s Quality via Multiple Syntactic Distance Evaluation Metrics

To assess the robustness and stability of the clustering algorithms under various conditions, we employed several approaches [38,39,40]. We computed three evaluation distance measures metrics (the SI, CHI, and DBI) for each algorithm (K-means, Gaussian mixture, and agglomerative clustering using all four linkage methods), and for each value of k, with and without the use of Gower’s distance metric. This allowed us to compare the performance of the different algorithms and examine the impact of the number of clusters (k) on the quality metrics.

#### 2.5.3. Iterative Phase

We tested the stability of our algorithms under different conditions, such as varying starting seeds, and using subsets of data. This helped us ensure the reliability of our clustering results.

##### Assessing the Clustering’s Sensitivity to Starting Seeds

We conducted a thorough evaluation to examine the impact of initial seeds on the performance of the K-means and Gaussian algorithms. To assess their sensitivity, we performed 30 iterations of each algorithm, both with and without the utilization of the Gower’s metric. This evaluation used various k values, employing SI, CHI, and DBI as the evaluation metrics. The results were presented in a box plot showcasing the mean score index across all runs. This analysis allowed us to assess the stability of algorithms under diverse starting conditions.

##### Within and between Clustering Consistency Using the Adjusted Rand Index (Ari)

We performed 30 iterations using a randomly selected subset amounting to 70% of the data to assess cluster consistency. We counted the number of “bad clusters” defined as clusters containing <5% of the data, and calculated the SI, CHI, and DBI scores for each algorithm and k value. The mean, standard deviation, and distribution of these scores were analyzed using box plots.

##### Within and between Clustering Consistency Using the Adjusted Rand Index (ARI)

To evaluate the overall clustering consistency, we applied each algorithm to the dataset for 10 iterations using random seeds. We saved the resulting labels after each iteration. Intra-algorithm consistency was assessed by calculating ARI scores for all possible pairs of labels (45 pairs in total for 10 iterations), assessing the consistency of patient assignment to the same cluster across different iterations, seeds, or metrics for each algorithm. Additionally, inter-algorithm similarity was examined by comparing the results of two different algorithms, aiming to verify the consistency of patient assignment with different algorithms.

##### Internal Semantic Assessment through Multiple Bagging Iterations Using Partial Subsets (70%) of the Data

In addition to the internal evaluation metrics and ARI scores, we conducted a semantic evaluation of the clustering results. For each k value, we clustered the dataset 10 times using a random 70% subset selected through bagging. To assign semantic labels to clusters across iterations, we manually identified semantically similar clusters based on key clinical features, to ensure that those with similar semantics had the same label. For example, we consistently labeled cluster “Ci,0” from iterations i = 1 to 10 as “Cluster 0”, which represented the cluster that appeared to be the “sickest” in each iteration. We identified cluster semantics using aggregative features, such as the sum of pain locations, and compared the proportions of categorical demographic and clinical features (e.g., percentage of females) among clusters with different semantics generated in different iterations. Specifically, we compared Cluster ${C\_(}_{i,k_{m}})$ (e.g., the semantically identified sickest cluster, generated for k = k, in iteration i) to Cluster ${C\_(}_{j,k_{m}})$ for 1 ≤ i, j ≤ 10, i ≠ j (e.g., the semantically identified sickest cluster generated in each of the 10 iterations). This comparison was performed for all m = 1..k clusters, resulting in 45 × k pairs of clustering instances being compared. We employed a Z proportion test to calculate the difference in proportion of each of the 151 categorical features for each cluster. This analysis helped us to assess the consistency of cluster semantics across iterations and identify potential sources of variability in the clustering results.

### 2.6. The SSV (Statistical and Semantic Validation) Phase

Once the method and the optimal number of k clusters were determined, we moved to the SSV phase. Here, we statistically validated the clusters’ clinical relevance by analyzing associations with various patient features.

#### 2.6.1. External (Clinical) Semantic Assessment Using Statistical Analysis

To statistically evaluate the selected clusters, we analyzed the associations of the clusters with continuous and categorical features. For continuous features, we calculated the mean and standard deviation and employed the student *t*-test (k = 2) or ANOVA corrected with Bonferroni (k > 2) to examine the differences in cluster distributions. For categorical features, we computed frequencies and percentages and utilized either Pearson’s chi-square test (k = 2) or the likelihood ratio test (k > 2). The significance level was set at 0.05 to determine the statistical significance of the observed results.

#### 2.6.2. Cluster Validation and Interpretation Using Machine Learning and SHAP

To validate the clusters identified, we used a random forest model to predict the cluster assignments for each patient. For this model, we utilized the default parameters as implemented in the scikit-learn (sklearn) library in Python. This machine learning approach was chosen for its robustness and ability to handle complex, multi-dimensional data. Further, to understand which features most influenced these predictions, we utilized SHAP values. SHAP values provide insights into the contribution of each feature to the prediction made by the model, thereby clarifying which features are most influential in defining each cluster, enhancing the interpretation of the clustering results. To facilitate this computation, we utilized the TreeExplainer method, designed for tree-based models [37,38]; like random forest, this method allows for an efficient and accurate interpretation of the model’s output. Moreover, to enhance interpretability, we grouped features into aggregative sums, enabling us to analyze the collective impact of related features on the clustering, providing a more holistic view of the factors that differentiated the patient clusters.

## 3. Results

### 3.1. Results of the CDI Phase

#### 3.1.1. Results of the Clustering Phase

**Figure A1:**
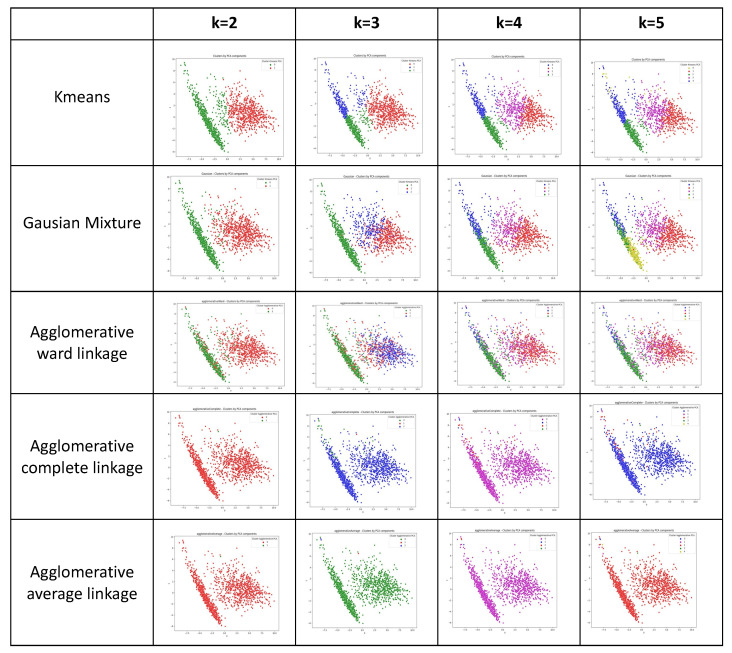
Visualization of different algorithms for different values of k. Each color represents a different cluster. The figure illustrates the clustering results obtained using different algorithms and values of k. Notably, the K-means and Gaussian clustering methods demonstrate a higher degree of similarity in their cluster assignments compared to the agglomerative clustering method utilizing the Ward linkage criterion. Also, it can be noted that when alternative linkage criteria were employed with the agglomerative clustering method, the majority of data points were assigned to a single cluster. This observation suggests that utilizing linkage criteria other than Ward may lead to less meaningful cluster assignments.

In total, 1370 subjects were included in the analysis. Initially, we employed principal component analysis (PCA) [41,42] to visualize the outcomes of various clustering algorithms across different k values. The PCA analysis incorporated 88 components, which accounted for over 80% of the data’s variance. For a visual representation of each algorithm across different k values, refer to Figure A1, Appendix A. The visualizations indicate that the K-means and Gaussian clustering methods exhibit greater similarity in their cluster assignments compared to those under the agglomerative clustering method using the Ward linkage criterion. Interestingly, applying different linkage criteria to the agglomerative method often resulted in most data points being assigned to a single cluster, suggesting that linkage criteria other than that of Ward may yield less meaningful cluster assignments.

**Figure A2:**
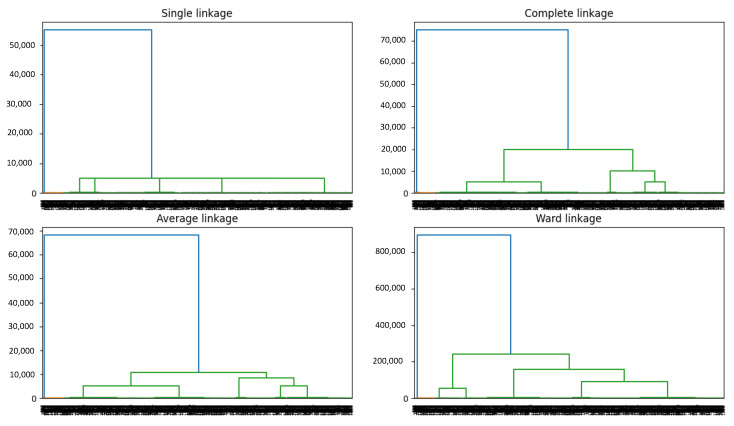
Dendrograms of hierarchical clustering using different linkage criteria. Each vertical line represents a merge between clusters. The height of the vertical lines represents the distance (or dissimilarity) at which clusters are merged. The colors signify different clusters formed, based on the standard threshold (70% of the maximum linkage distance).

Additionally, we examined the impact of various linkage criteria on agglomerative clustering results as illustrated by a dendrogram in Figure A2, Appendix A. The dendrogram reinforces our observation that employing linkage criteria other than that of Ward tends to result in less meaningful cluster assignments. Consequently, the careful selection of an appropriate linkage criterion is crucial for achieving meaningful results in agglomerative clustering.

#### 3.1.2. Results of the Distance Measure Phase

**Figure 2:**
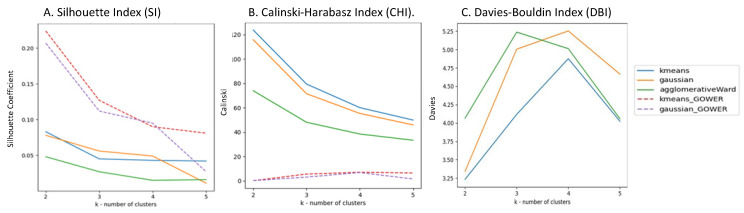
Evaluation of clustering algorithms using evaluation metrics. (**A**) Silhouette index (SI): the SI scores for different values of k indicate that K-means with Gower’s distance metric achieved the highest score for k = 2, 3, and 5. (**B**) Calinski–Harabasz index (CHI): K-means consistently outperformed other algorithms, achieving the best score across all values of k. (**C**) Davies–Bouldin index (DBI): K-means demonstrated superior results for all values of k.

The evaluation metrics (SI, CHI, and DBI) were employed to assess the quality of clusters generated by the K-means, Gaussian mixture, and agglomerative Ward algorithms for various k values. These results are depicted in Figure 2.

**Figure A3:**
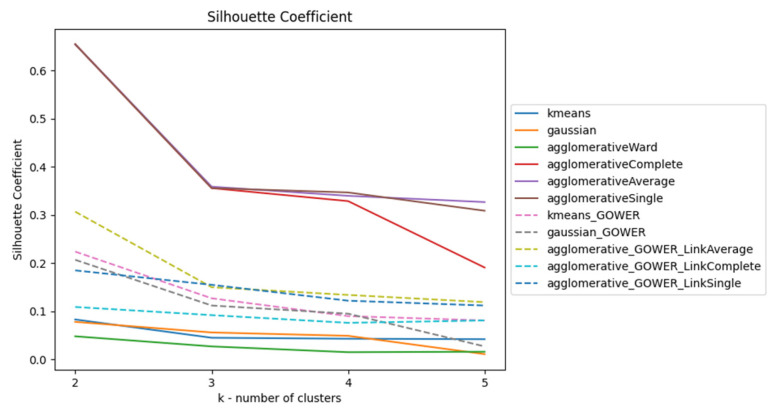
The SI scores of each algorithm in the different values of k. The agglomerative algorithm with complete, average and single linkage had the best scores for every k. The agglomerative algorithm with ward linkage was the worst for almost every k (except k = 5). K-means and Gaussian mixture have very similar scores, both when using Gower’s distance metric and when not using it.

**Figure A4:**
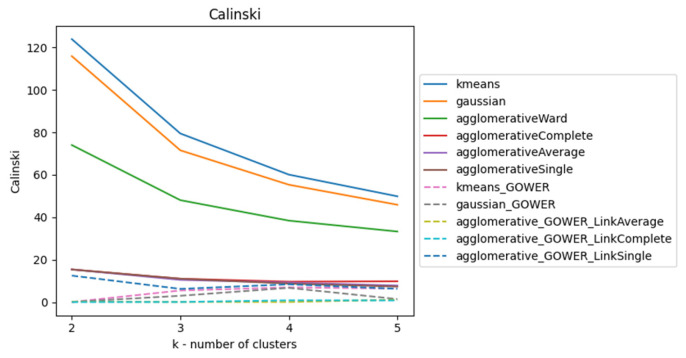
The CHI score of K-means was superior for every k, followed by Gaussian mixture and the agglomerative algorithm with ward linkage.

**Figure A5:**
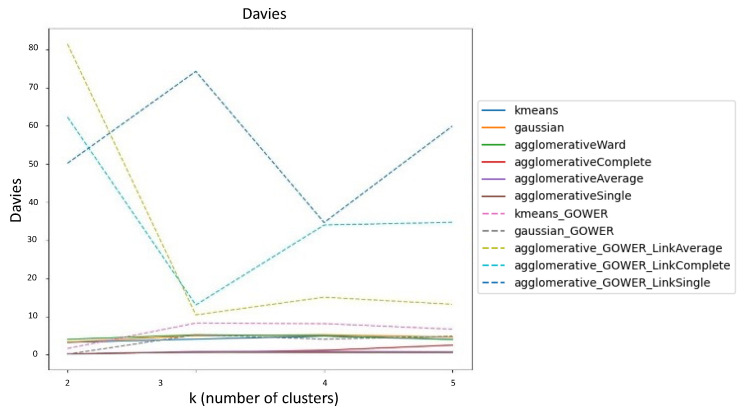
On the left, the DBI scores of all algorithms; on the right, after the removal of the three worst (highest) ones. The agglomerative algorithm with complete, average, and single linkage had the lowest (best) results for all ks. K-means performed better than Gaussian mixture did for all ks. Gaussian mixture performed better than did the agglomerative algorithm with Ward linkage for k = 2 and 3 but not for k = 4 and 5.

For clarity, we omitted results from agglomerative algorithms with linkages that clustered almost all points into a single cluster. However, their results can be found in Figure A3, Figure A4 and Figure A5, Appendix A.

##### Silhouette Index (SI)

The SI measure displayed in Figure 2A shows that using Gower’s distance metric improved the results. Specifically, K-means with Gower’s distance metric achieved the highest SI score for k = 2, 3, and 5, followed by Gaussian mixture with Gower’s distance metric, which exhibited a slightly better score for k = 4. The agglomerative Ward algorithm performed relatively worse across most k values. Additionally, K-means outperformed Gaussian mixture for k = 1 and 5 but not for k = 2 and 3. Notably, the SI score tends to decline with an increasing k value in almost all algorithms, except for K-means, where it remains relatively consistent for k = 3, 4, and 5.

##### Calinski–Harabasz Index (CHI)

Figure 2B illustrates the CHI measure. K-means consistently achieved the highest score for all k values, followed by Gaussian mixture and the agglomerative algorithm with Ward linkage. Interestingly, the use of Gower’s metric led to inferior results. The CHI score also decreased as k increased.

##### Davies-Bouldin Index (DBI)

The DBI measure is depicted in Figure 2C. The use of Gower’s metric significantly worsened the results, leading to their exclusion from Figure 2C. K-means consistently attained the best (lowest) DBI score across all k values. Gaussian mixture outperformed the agglomerative algorithm with Ward linkage for k = 2 and k = 3 but not for k = 4 and k = 5. Unlike the SI and CHI scores, no improvement in the DBI score was observed as the k increased.

In summary, Figure 2 shows that K-means outperformed the other algorithms in two of the three evaluation metrics. Specifically, in terms of the CHI score, K-means demonstrated superior performance across all k values, surpassing all other algorithms. Additionally, for the DBI score, K-means achieved the best (lowest and thus best) score across all k values after excluding algorithms that clustered most points into a single cluster. These results suggest that K-means exhibits greater robustness and stability compared to those of the other algorithms examined in our study.

#### 3.1.3. Results of the Iterative Phase

##### Assessment of Clustering Algorithms’ Sensitivity to Initial Seeds

**Figure A6:**
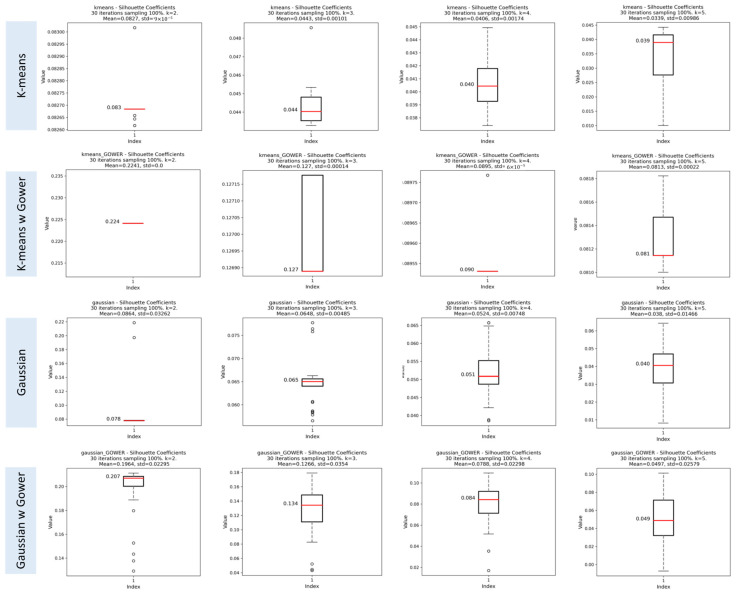
Distribution of SI scores for K-means and Gaussian with and without Gower’s distance metric for the different values of k, sampling 100% of the data, when using different starting seeds.

**Figure A7:**
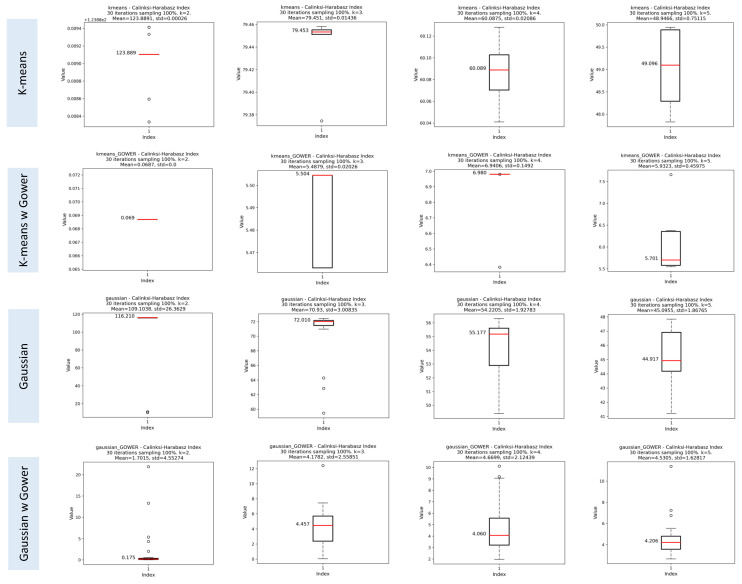
Distribution of CHI scores for K-means and Gaussian mixture with and without Gower’s distance metric for the different values of k, sampling 100% of the data, when using different starting seeds.

**Figure A8:**
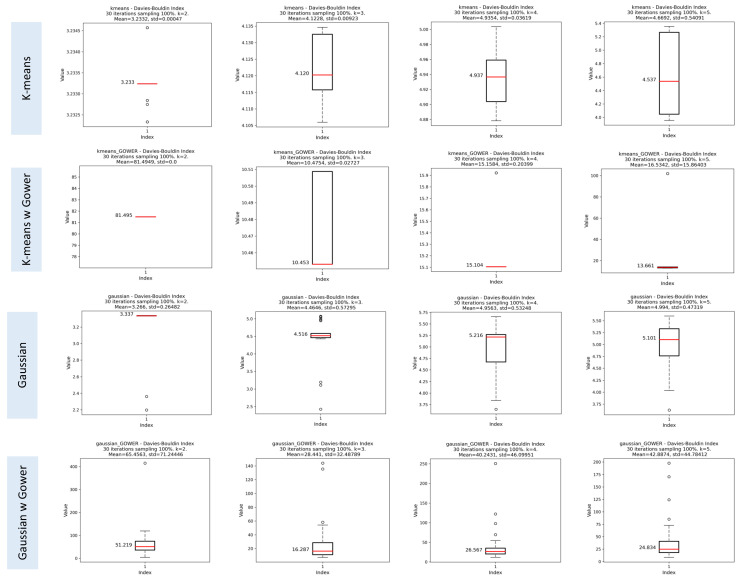
Distribution of DBI scores for K-means and Gaussian mixture with and without Gower’s distance metric for the different values of k, sampling 100% of the data, when using different starting seeds.

We conducted 30 iterations of the K-means and Gaussian algorithms, with varying starting seeds for different k values. As expected, agglomerative clustering was not influenced by the starting seed. The results are depicted in Figure A6, Figure A7 and Figure A8, Appendix A, which present the boxplots of SI, CHI, and DBI scores.

Although clustering algorithms are acknowledged to be sensitive to initial seeds, we found minimal variation in performance across different seeds in our dataset. K-means with k = 2, 3, and 4 exhibited a standard deviation of performance of less than 0.05 across seeds. However, using Gower’s metric led to increased variance in certain cases, yielding inferior results in terms of the DBI score. Hence, our findings suggest that while Gower’s metric can improve performance and reduce variance in some scenarios, it might increase variance in others.

##### Evaluation of Cluster Consistency Using Random Subsets of 70% of the Data

In this assessment, involving counting the number of “bad clusters”, K-means, both with and without the Gower metric, did not generate any bad clusters for k = 2, 3, and 4. Conversely, agglomerative algorithms using average, single, and complete linkages consistently generated a high number of bad clusters, as detailed in Table A1, Appendix A. These findings are supported by the visualization in Figure A1, where these algorithms clustered most points into a single cluster, resulting in underrepresented clusters. Using the Gower metric in K-means, Gaussian mixture, and agglomerative clustering with complete linkage reduced the number of bad clusters and improved the clustering iterations. Notably, an increase in the value of k corresponded to a proportional rise in the number of bad clusters across all algorithms.

##### Comparison of SI, CHI, and DBI Scores using 100% and 70% of Data

**Figure A9:**
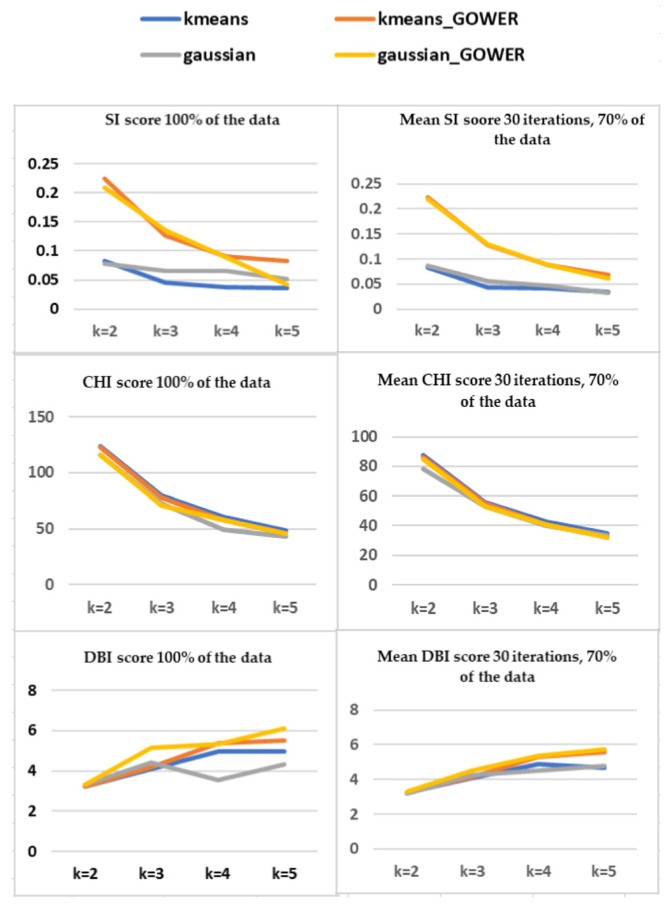
Comparison of silhouette index (SI), Calinski–Harabasz index (CHI), and Davies–Bouldin index (DBI) scores using 100% (**left**) and 70% (**right**) of the data. The figure shows consistent performance between 70% and 100% of the data, as mean scores did not significantly vary between them.

To explore clustering performance, we calculated the SI, CHI, and DBI scores for each iteration and k value, using a random subset of 70% of the data, and compared them to the scores obtained when using the complete dataset. The comparative analysis, presented in Figure A9, Appendix A, reveals consistent performance, with mean scores showing little variation between using 100% and 70% of the data.

##### Assessing Consistency within and between Clustering Methods Using the Adjusted Rand Index (ARI)

To evaluate consistency within and between clustering algorithms, we conducted an analysis of intra-algorithm and inter-algorithm similarity. For each algorithm and k value, we performed clustering on the entire dataset using 10 random seeds, saved the resulting labels, and calculated the ARI score for all possible pairs of labels, resulting in 45 pairwise comparisons. The results of the intra-algorithm and inter-algorithm analyses are presented in Table 1.

In the **intra-algorithm similarity analysis**, K-means demonstrated remarkable robustness, with minimal differences observed for k = 2 and k = 3. The utilization of Gower’s metric improved the algorithm’s robustness across all k values. Interestingly, both K-means and Gaussian mixture produced highly similar clustering results, regardless of whether they used Gower’s distance metric or not, with ARI scores of 0.944 and 0.978, respectively. As expected, the agglomerative algorithm was unaffected by different seeds and consistently yielded identical results, resulting in an intra-score of 1. The ARI scores of K-means with different metrics were quite similar, particularly for k = 2 and k = 3 (0.944 and 0.819 respectively). Similarly, Gaussian mixture with different metrics also achieved a very high score for k = 2 (0.978).

The **inter-algorithm similarity analysis** revealed a high ARI between K-means and Gaussian mixture for both metrics. Interestingly, when both algorithms employed the Gower metric, the ARI increased for k = 4 and k = 5 (0.931 and 0.900 respectively). The agglomerative algorithm with Ward linkage also exhibited a high ARI score, while the agglomerative algorithm with other linkages demonstrated lower similarity.

### 3.2. Results of the SSV Phase

#### 3.2.1. The Semantic Phase

##### Semantic Assessment of Clustering Methods Using 70% of the Data

Following the internal evaluation metrics and ARI score, we conducted a semantic assessment of the clustering algorithms using subsets comprising 70% of the data. K-means was chosen due to its superior performance in previous assessments, evidenced by its CHI and DBI scores, robustness in intra-algorithm analysis, and similarity to Gaussian mixture and agglomerative (Ward) algorithms in the inter-algorithm analysis. K-means generated no bad clusters for k = 2, 3, and 4, but had a few bad clusters for k = 5. Despite the known influence of the starting seed, we noted minimal variability in the scores across different seed runs.

To conduct this analysis, we clustered the dataset 10 times using random subsets of 70% of the data for each k value. In each iteration, we manually relabeled clusters. We then conducted Z proportion tests to compare demographic and clinical categorical features between clusters with different semantics.

For k = 2 and k = 3, no statistically significant differences were observed between any pair of clusters at an alpha level of 0.001, indicating semantic consistency even with 70% of the data. For k = 4 and k = 5, we found statistically significant differences in 55 pairs and 2822 pairs, respectively, at alpha = 0.001.

##### External Semantic Assessment Using Statistical and Clinical Evaluation of Selected Clusters

Although both k = 2 and k = 3 were viable syntactic solutions for K-means, our semantic statistical analysis indicated that k = 3 held more clinical significance. Therefore, we will detail the k = 3 clusters generated by K-means in the following paragraphs. The results for k = 2 are included in Appendix A and discussed below.

##### Demographics and Smoking Habits across the Clusters

**Figure 3:**
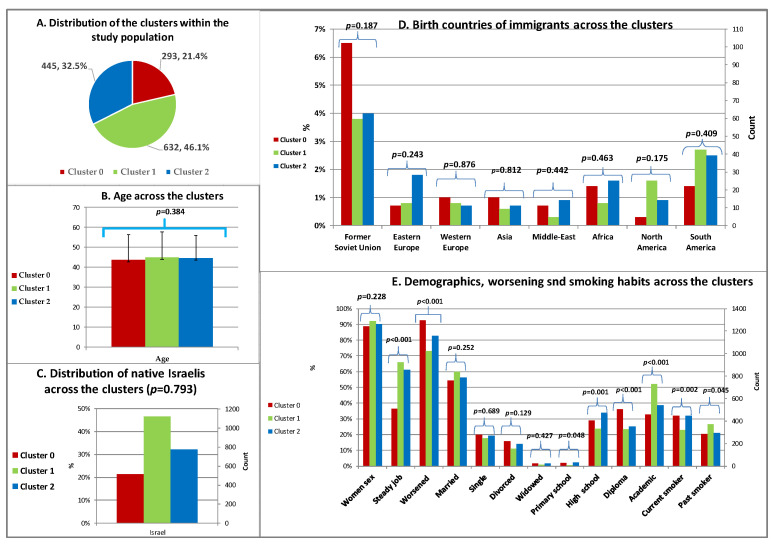
Demographics and smoking habits across the clusters (k = 3) (likelihood ratio).

The age range was 8–85, the mean age was 44.5 ± 12.4 years, and 1243 (90.7%) of the participants were women while 127 (9.3%) were men. The demographics and smoking habits across the clusters are presented in Figure 3.

The distribution of the clusters within the study population was as follows: Cluster 0 (293 subjects, 21.4%), Cluster 1 (632, 46.1%), and Cluster 2 (445, 32.5%) (Figure 3A).

No statistically significant associations were found between any specific cluster and the following demographic characteristics: age (*p* = 0.384, Figure 3B), sex (*p* = 0.228, Figure 3E), being native Israeli (*p* = 0.793, Figure 3C), being born in any other immigrant countries (Figure 3D), and marital status (Figure 3E). However, significant differences were observed among the clusters in relation to other factors. As depicted in Figure 3E, Cluster 1 reflected the least severe condition, Cluster 0 reflected the worst, and Cluster 2 fell in between. The following comparisons showed statistically significant differences among the clusters: having a steady job (*p* < 0.001), reporting a worsening of fibromyalgia in the last year (*p* < 0.001), and current smoking status (*p* < 0.001). Cluster 0 had the highest prevalence among those with a high school education (*p* = 0.001) and diploma education (postgraduate qualification after high school, but not an academic degree) (*p* < 0.001).

##### Comorbidities and History of Trauma across the Clusters

**Figure 4:**
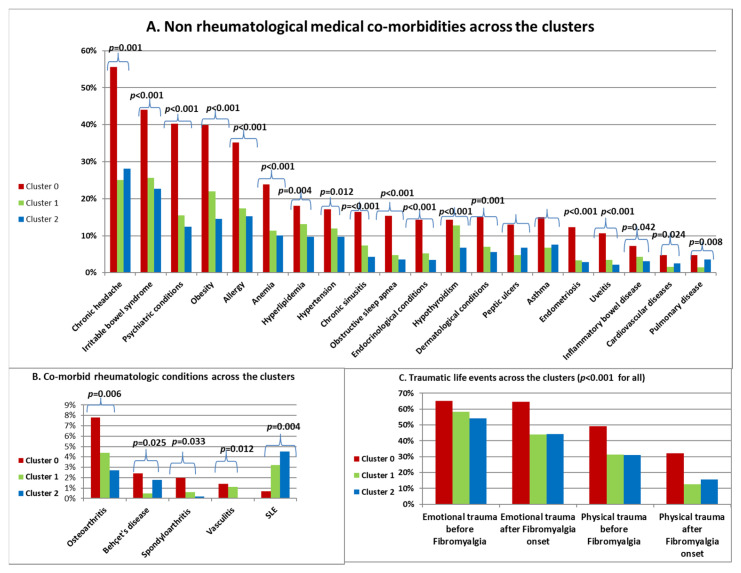
Comorbidities and history of trauma across the clusters (likelihood ratio).

The distribution of comorbidities and trauma history across the clusters are presented in Figure 4. Cluster 0 had a significantly higher prevalence of all analyzed systemic diseases (Figure 4A), as well as of rheumatological conditions, except for systemic lupus erythematosus (SLE), where it showed a significantly lower prevalence (Figure 4B). Additionally, Cluster 0 exhibited a higher number of emotional and physical traumatic life events both before and after the onset of fibromyalgia (Figure 4C). There were no statistically significant differences observed between the three clusters regarding the presence of certain systemic comorbidities, including malignancy (*p* = 0.619), hyperthyroidism (*p* = 0.194), liver disease (*p* = 0.086), and kidney disease (*p* = 0.921). Similarly, no significant differences were found among the clusters for various comorbid rheumatological conditions, including rheumatoid arthritis (*p* = 0.209), Sjögren syndrome (*p* = 0.977), Ankylosing spondylitis (*p* = 0.155), psoriatic arthritis (*p* = 0.073), familial mediterranean fever (*p* = 0.587), scleroderma (*p* = 0.307), gout (*p* = 0.074), and pseudogout (*p* = 0.214); these non-significant findings are not shown in the figures.

##### Symptoms, Sleep and Functional Problems and Treatment Modalities across the Clusters

**Figure 5:**
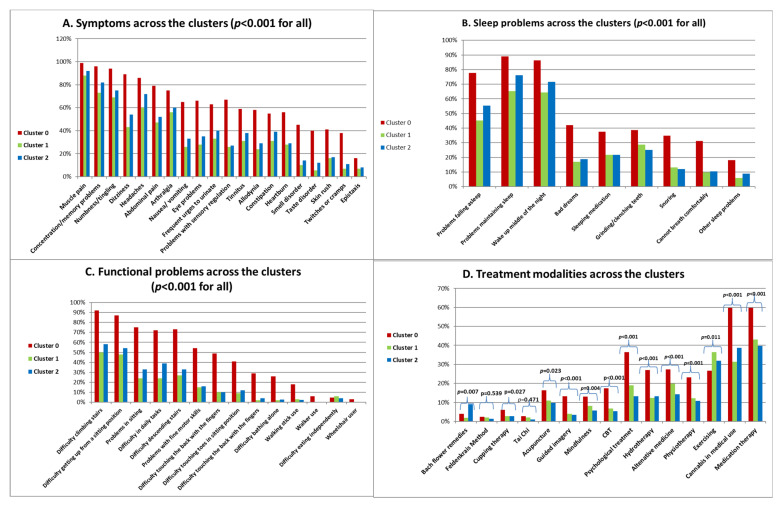
Symptoms, sleep and functional problems and treatment modalities across the clusters (likelihood ratio).

The distribution of symptoms, sleep problems, functional mobility problems, and treatment modalities across the clusters are presented in Figure 5. Cluster 0 exhibited a significantly higher number of symptoms (Figure 5A) along with a greater prevalence of sleep problems (Figure 5B) and functional mobility issues (Figure 5C). Regarding treatment modalities, Cluster 0 underwent more treatments overall, except for exercising (*p* < 0.001). Notably, no significant differences were observed in the use of certain treatments, such as the Tai Chi (*p* = 0.256) and Feldenkrais method (*p* = 0.539) (Figure 5D).

##### Years with Fibromyalgia, Pain, Sleep, Quality of Life and Treatment Effectiveness across the Clusters

**Figure 6:**
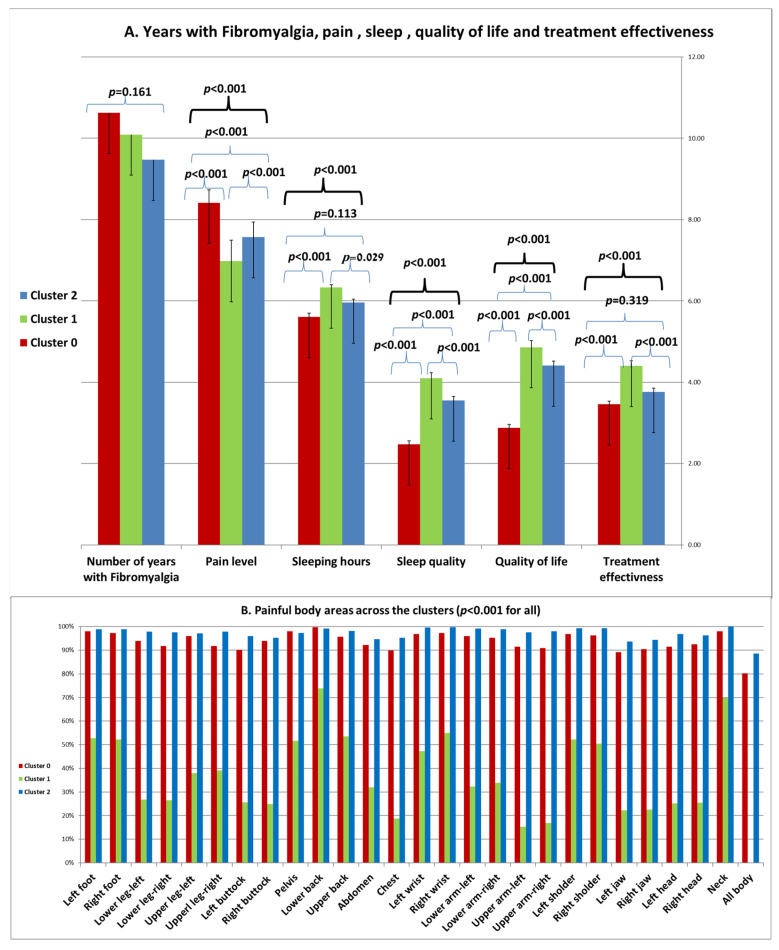
Years with fibromyalgia, sleep, quality of life, treatment effectiveness, and pain level and locations (analysis of variance (ANOVA) corrected with Bonferroni test for multiple comparisons).

The ANOVA analysis and post hoc Bonferroni tests examining the years with fibromyalgia, pain levels, sleep, quality of life, and treatment effectiveness across the clusters is presented in Figure 6. The number of years patients had fibromyalgia did not show any statistically significant differences between the clusters (*p* = 0.161). As illustrated in Figure 6A, Cluster 0 represents the most severe condition, Cluster 1 represents the least severe condition, and Cluster 2 falls in between. Significant differences were observed among the clusters in terms of pain levels, sleeping hours, sleep quality, and quality of life. Cluster 0 reported the lowest scores in treatment effectiveness, which were statistically significantly lower than those of Cluster 1 (*p* < 0.001), but not statistically significant compared to those of Cluster 2 (*p* = 0.319).

The distribution of specific pain locations across the clusters is depicted in Figure 6B. Statistically significant differences were observed between the clusters for all body locations. Contrary to previous observations, the highest proportions of patients reporting pain were found in Cluster 2, followed by Cluster 0, which exhibited similar proportions in all painful areas. Cluster 1 had the lowest proportions of patients reporting pain in various body areas. Notably, none of the patients in Cluster 1 reported pain in all body areas.

In summary, our statistical and clinical evaluation of the k = 3 clusters indicates that Cluster 0 represents the most severe condition, Cluster 1 represents the least severe condition, and Cluster 2 falls in between. Significant differences were observed among the clusters in terms of comorbid medical conditions, symptoms, sleep patterns, functionality, and treatment outcomes. However, no significant differences were observed in terms of pain locations.

#### 3.2.2. The Validation Phase: A Cluster Classification Model and Computation of Its SHAP Values to Assess the Relative Importance of Different Features When Forming Clusters

We validated the clustering results using a random forest model to predict cluster assignments, incorporating aggregated features like medical comorbidities and treatments. We obtained a mean ROC (receiver operating characteristic) AUC (area under the curve) score of 0.9943 and an overall accuracy of 0.9459 with 10-fold cross-validation. To assess the relative importance of these aggregated features in predicting and interpreting the clusters, we calculated SHAP values.

**Figure 7:**
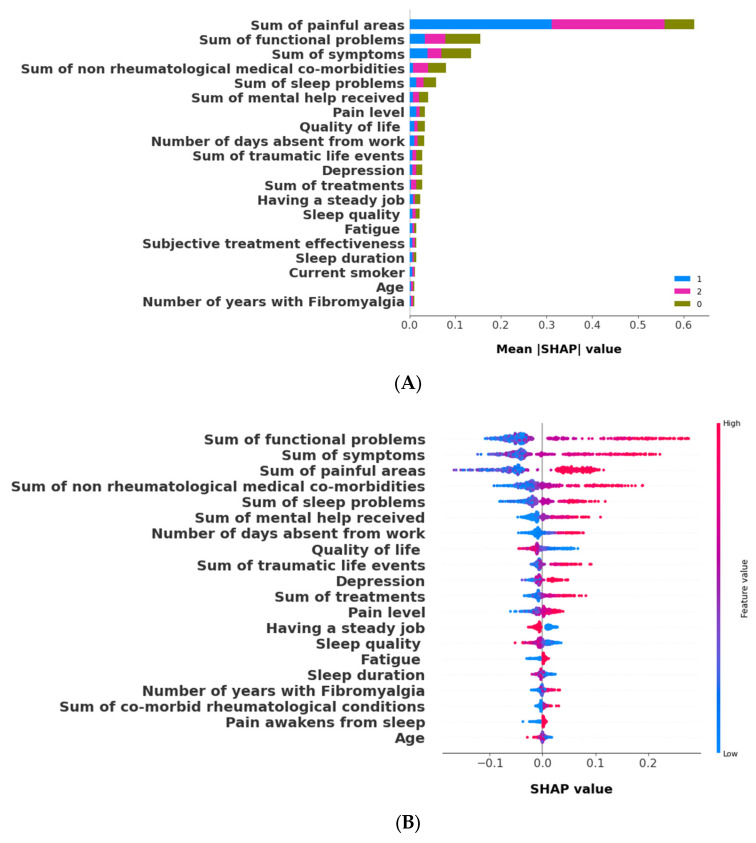

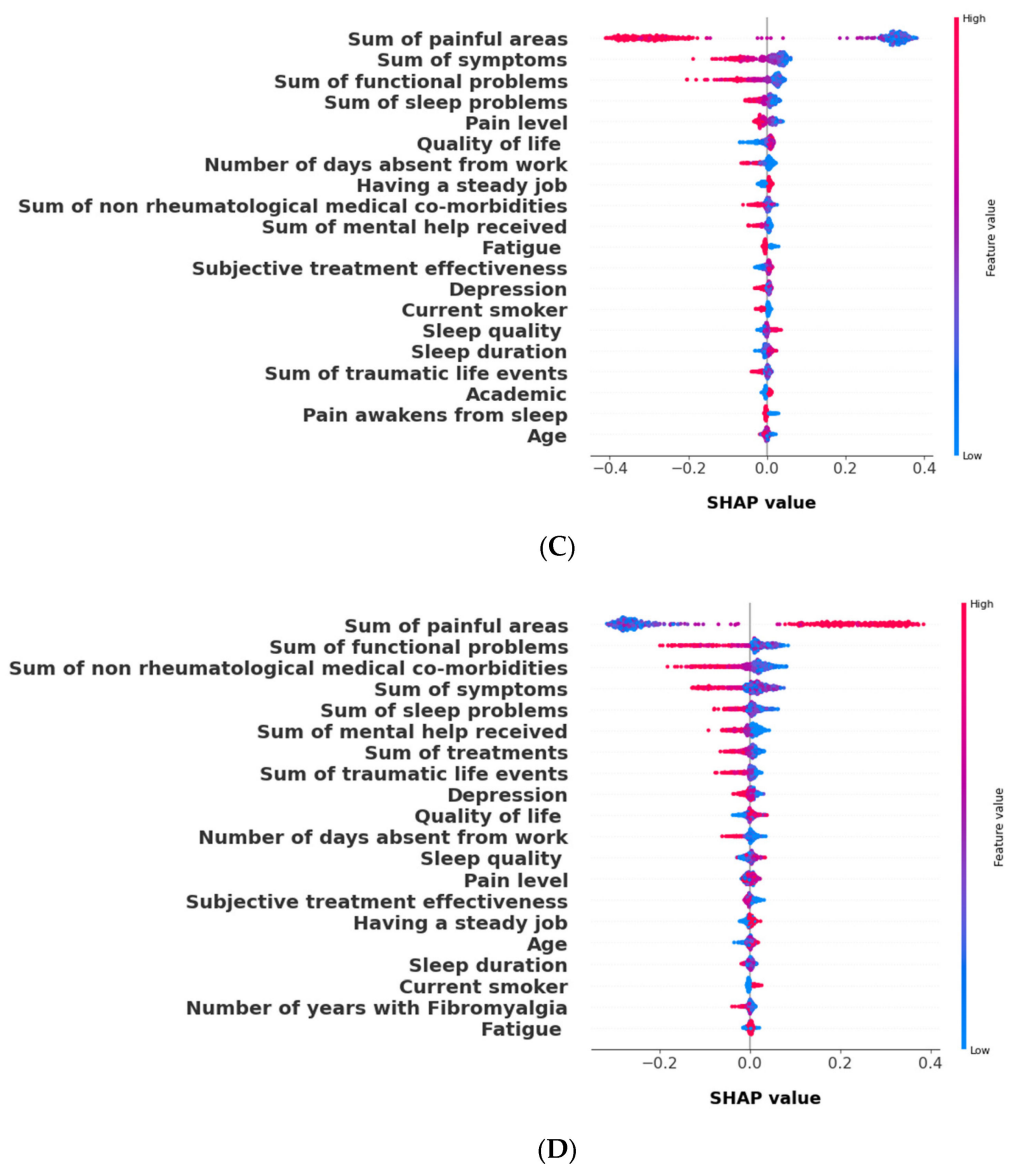
SHAP (Shapley additive explanations) model to predict clusters, k = 3. (**A**) Bar plot for mean SHAP values of k = 3; (**B**) dot plot for Cluster 0; (**C**) dot plot for Cluster 1; (**D**) dot plot for Cluster 2.

Figure 7A displays the top 20 features in the cluster prediction. Dot plots for cluster 0, 1, and 2 are presented in Figure 7B–D, respectively. Figure 7B shows that Cluster 0 (sickest) was uniquely positively associated with mobility functional problems, the most significant feature for this cluster. In contrast, Cluster 1 (healthiest) and Cluster 2 ranked the sum of painful areas as the most significant parameter and exhibited a negative association with mobility problems, as depicted in Figure 7C,D, respectively. While Cluster 0 and 2 were positively associated with the sum of painful areas, Cluster 1 demonstrated a negative association. Cluster 0 also had positive associations with several symptoms, painful areas, comorbidities, sleep problems, mental health, and work absence, but showed negative associations with quality of life, steady employment, and sleep quality and duration. Age did not significantly contribute to cluster differences.

### 3.3. The k = 2 Solution

The k = 2 solution represented valid syntactic clustering, as determined by the three-distance metrics used. However, both k = 2 and k = 3 were legitimate syntactic solutions according to the ARI stability metric. Therefore, we conducted a semantic statistical analysis to assess the clinical relevance of the clusters for both k = 2 and k = 3. The k = 3 solution emerged as a meaningful form of clustering, identifying three sub-classes of fibromyalgia severity: Cluster 0 (most severe condition), Cluster 1 (least severe condition), and Cluster 2 (intermediate). Significant differences were observed in various comparisons related to comorbid medical conditions, symptoms, sleep patterns, functionality, and treatment outcomes, although not in terms of pain locations.

To evaluate the k = 2 solution, we employed the same statistical tests, ML model (random forest), and SHAP explanations. Detailed results for the k = 2 solution are available in the Appendix A. k = 2 clustering resulted in two clusters: Cluster 0 with 731 subjects and Cluster 1 with 639 subjects. Cluster 0 consisted of patients with more severe conditions, while Cluster 1 comprised patients with less severe conditions. Further analysis showed that Cluster 0 in the k = 2 solution combined elements of both Cluster 0 (most severe) and Cluster 2 (intermediate severity) from the k = 3 solution. Patients in Cluster 1 for the k = 3 solution predominantly remained as Cluster 1 (healthier cluster) in the k = 2 solution.

To assess differences within each feature between the two clusters, we used Pearson’s chi-square test for categorical parameters and an independent *t*-test for continuous variables. The results of these tests are detailed in Table A2 in Appendix A. Although there were differences between the two clusters in the k = 2 solution, the k = 3 solution exhibited a greater number of statistically and clinically significant features. The absence of significant differences in certain features could be attributed to the merging of the most severe and intermediate clusters.

**Figure A10:**
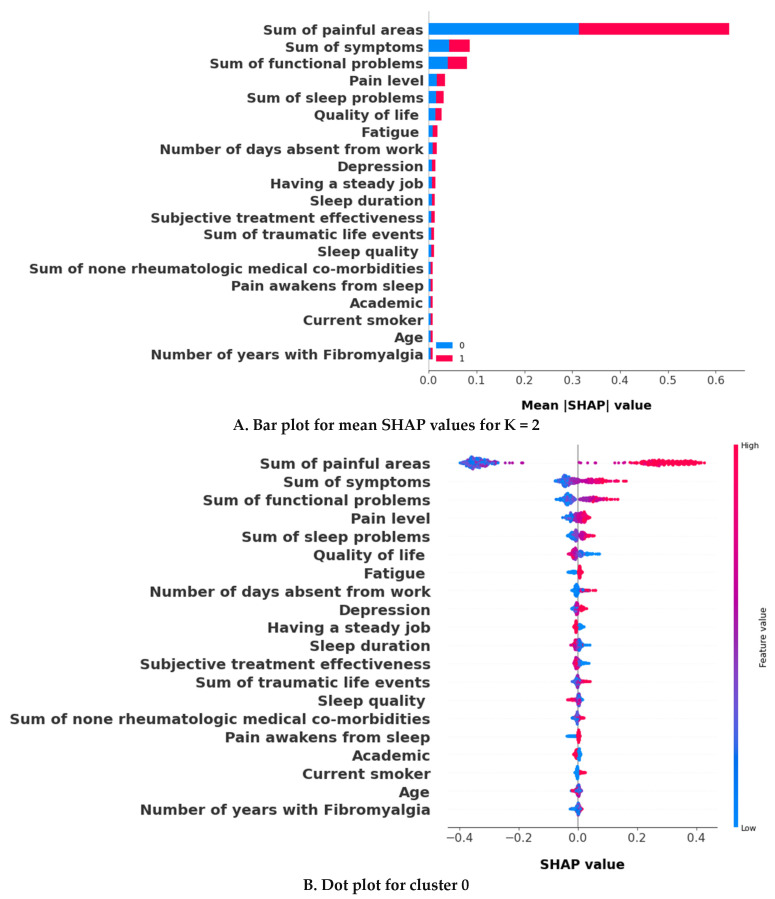

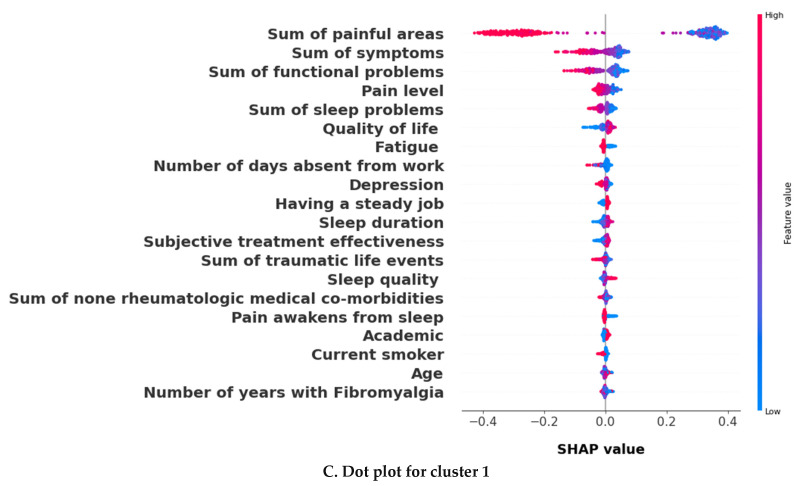
SHAP (SHapley Additive exPlanations) Model to predict clusters for K = 2.

In the prediction models for k = 2 using the random forest model with 10-fold cross-validation, we achieved an ROC AUC and accuracy of 0.99. The SHAP algorithm results for K-means clustering with k = 2 are presented in Appendix A (Figure A10). Pain locations and mobility functional problems were highly ranked in both Cluster 0 and Cluster 1, but with opposite associations.

Both k = 2 and k = 3 partitioning options using K-means are valid clustering solutions. However, the k = 3 solution holds greater clinical significance and may contribute to a better understanding of the underlying mechanisms of fibromyalgia, potentially leading to more effective therapeutic interventions. Therefore, both solutions are presented in the results of our study.

**Figure 8:**
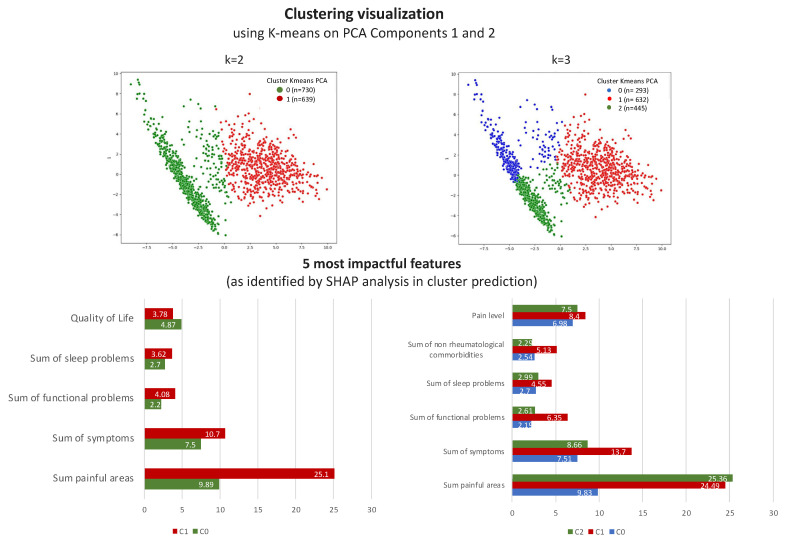
**Comparative visualization of k = 2 and k = 3 clustering solutions in fibromyalgia patient analysis.** The top-left panel displays k = 2 clustering using PCA components, clearly delineating Clusters 0 and 1. The top-right panel presents k = 3 clustering, offering a detailed view of Clusters 0, 1, and 2. The bottom panel includes bar plots that highlight the five most significant attributes for each cluster, with the left side pertaining to the k = 2 solution and the right side pertaining to the k = 3 solution.

To better understand the nuanced differences and key characteristics that distinguish the k = 2 and k = 3 clustering solutions, we included Figure 8. This figure displays the cluster visualizations as defined by the k-means algorithm for both k = 2 and k = 3 scenarios, using the first two PCA components. This approach provides a more instinctive comprehension of the clusters’ structure and the critical factors differentiating them. Additionally, the figure includes bar plot graphs that highlight the top five influential features for each cluster, as identified through our SHAP analysis. These bar plots provide insights into the defining characteristics of each patient group, thereby enhancing our understanding of each cluster in the context of fibromyalgia.

## 4. Discussion

The present study introduces the CDI-SSV methodology, a novel multi-dimensional approach to discover and validate the optimal number of clusters. Unlike traditional clustering approaches that often rely on a single algorithm or metric, our method uniquely integrates several **clustering** algorithms, **distance** measures, and bagging and clustering **iterations** (the **CDI** phase), followed by the **SSV** phase, computing **statistical** differences among clusters for several meaningful additional clinical **semantic** features. Finally, to validate our results, we generated a machine learning model that classified the patients into clusters and assessed the importance of the demographic and clinical features, using SHAP values.

A key innovation of our study is the application of this multi-dimensional approach to a large cohort of 1370 fibromyalgia patients, a scale significantly larger than that of most previous studies in this domain. This extensive sample size allows for the capturing of a broader spectrum of patient variability, thereby enhancing the reliability and applicability of our findings.

To the best of our knowledge, this is the first study published that employs such a holistic and multi-dimension methodology in a medical context, demonstrated here with fibromyalgia patients. The integration of multiple clustering algorithms alongside both syntactic and semantic validation techniques sets our approach apart from existing methods. Furthermore, the incorporation of SHAP values in the validation process not only provides a deeper understanding of the influence of demographic and clinical features on cluster formation but also highlights the potential of our methodology in the realm of personalized medicine.

We suggest that the CDI-SSV methodology can be effectively applied in across various medical domains for clustering analysis to identify patient sub-groups. Its capability to handle large datasets and integrate multiple data dimensions makes it a versatile tool for uncovering meaningful patterns in complex medical data. This approach has the potential to significantly contribute to the advancement of patient stratification and personalized treatment strategies, extending well beyond the scope of fibromyalgia to other medical conditions.

The present study found K-means to be a more robust and stable clustering method compared to other algorithms tested. This was noted due to several findings. First, our results, presented in Figure 2, indicated that K-means outperformed other algorithms in two out of three evaluation metrics (CHI and the DBI scores). K-means with Gower’s distance metric also had the best SI score for k = 2, 3, and 5. Moreover, we conducted 30 iterations of K-means and Gaussian mixture clustering algorithms with different seeds to assess their average performance. Figure A6, Figure A7 and Figure A8 in Appendix A show the results. Our findings demonstrate that the variation in performance across different seeds was minimal, especially for K-means with k = 2 and 3, in our dataset. Furthermore, we conducted 30 iterations using randomly bagged subsets (selected with replacement) comprising 70% of the data, and the results presented in Table A1 demonstrate that K-means did not create “bad” clusters, defined as clusters with less than 5% of the data, for k = 2, 3, or 4 with or without using the Gower distance metric. The mean SI, CHI, and DBI scores did not vary significantly when using 100% compared to 70% of the data (Figure A9). The assessment of the consistency within and between clustering methods using the adjusted Rand index (ARI) revealed that again, K-means was found to be a very robust algorithm, which was able to cluster individuals similarly to Gaussian mixture and agglomerative (Ward) algorithms with almost no difference in the ARI for k = 2 and k = 3. Based on the best overall performance of K-means according to all these assessments, we chose K-means as the preferred method and performed a semantic assessment of clustering methods using 70% of the data. No statistically significant differences were found, across all 151 categorical features, between any pair of equivalent clusters for k = 2 and k = 3 at a significance level of alpha = 0.001. Considering that both K = 2 and k = 3 were legitimate syntactic solutions, we further performed statistical analysis and evaluated the clinical relevance of the created clusters. While a cluster number of k = 2 yielded better syntactic performance in the SI, CHI, and DBI scores, the ARI scores of k = 2 were similar to those of k = 3, suggesting that even with a larger number of clusters, stability is maintained with respect to the same pairs of patients appearing in the same cluster. Even more importantly, k = 3 partitioning seemed to represent a more clinically meaningful partition, since the three clusters’ solution better explained the clinical picture presented by fibromyalgia patients, which seems to be composed of low-, intermediate-, and high-grade severity patients. Compared to the k = 2 solution, the k = 3 solution manifested more statistically significant differences in all comparisons among clusters in terms of comorbid medical conditions, symptoms, sleep patterns, functionality, and treatment outcomes, but not in terms of pain locations.

A recent study by Fernández-de-las-Peñas et al. [10] also found differences between subgroups of fibromyalgia patients in terms of psychological, cognitive, health-related, and physical features but similar widespread pressure pain sensitivity. However, their study, which identified only two subgroups, had a smaller population size (113) compared to that of our study (1370) and included only women. Additionally, their methodology differed from ours, as we employed a detailed CDI method to assess clustering. Finally, in our study, the sickest cluster was the smallest, representing 21.4% of the population, which may be challenging to capture in smaller cohorts.

Widespread pain is the hallmark of fibromyalgia, and therefore may not discriminate well between fibromyalgia patients. Fibromyalgia is now thought to be a pain regulation disorder, often classified as central sensitization [6], due to alterations in central nervous system pain and sensory processing [7]. We found differences between clusters not only in subjective parameters, but also in objective parameters, such as the presence of systemic and rheumatological comorbidities, symptoms, and functional problems such as using a walking stick or a wheelchair, which indicates a more serious clinical condition. These comorbid conditions may also contribute to pain. Therefore, the clinical implications of identifying these subgroups could imply different underlying mechanisms in each of these subgroups, a hypothesis that should be studied in future research.

Finally, to validate our clustering results using a supervised classification methodology, our random forest model accurately classified patients into three clusters with an AUC of 0.994 and accuracy of 0.946. Then, by computing the model’s SHAP values, we identified a distinct profile that enabled the model to classify the patients into each cluster. In particular, Cluster 0, the sickest cluster, is characterized by mobility functional problems, accompanying symptoms, painful areas, comorbidities, sleep and mental health problems, absenteeism, a lower quality of life, and treatment effectiveness self-assessment. These features serve as markers for evidence-based personalized diagnosis and might suggest that this subgroup requires different management strategies, providing clinical application points to patient-centered treatment.

The identification of three distinct fibromyalgia patient profiles in our study, as shown in Figure 8, has important implications for clinical management. These profiles enable more personalized treatment strategies, allowing clinicians to tailor interventions to each subgroup’s severity and characteristics. For example, the most severely affected cluster may require aggressive, multidisciplinary treatment, while others could benefit from less intensive therapies focused on lifestyle and symptom management. These findings also inform future research into fibromyalgia’s pathophysiology, particularly in understanding different patterns of central sensitization across subgroups. This knowledge is crucial for developing targeted therapies. Applying our CDI-SSV methodology in clinical practice can facilitate the early identification of patient subgroups, leading to earlier, more effective interventions and potentially better long-term outcomes. Ultimately, our study’s insights could significantly refine fibromyalgia diagnosis, management, and treatment, aligning with personalized medicine principles and improving patient care.

**Strength and limitations: ** The main contributions of the study include [1] a novel, highly general, multidimensional clustering methodology, CDI-SSV, for identifying patient subgroups; [2] the application of the CDI-SSV methodology to a dataset of fibromyalgia patients, which demonstrated its effectiveness in uncovering three distinct patient profiles, enabling a more nuanced understanding of fibromyalgia based on demographic and clinical features, and providing a potential to improve clinical care. The provision of clinical markers for evidence-based personalized diagnosis, management, and prognosis enables a more personalized tailoring of treatments and interventions.

Regarding limitations, although this study analyzed important features, it would also be useful to obtain genetic and laboratory results, thus enabling us to better understand the clinical significance of the different clusters.

## 5. Conclusions

In conclusion, our study highlights the value of the CDI-SSV methodology in clustering and classifying fibromyalgia patients, demonstrating its potential applicability beyond fibromyalgia to other medical domains. This methodology facilitates enhanced patient stratification, paving the way for improved clinical outcomes across various conditions. The identification of distinct profiles within fibromyalgia patients allows for a more targeted and personalized approach in diagnosis, management, and prognosis. The practical implications of these findings, including the potential for more effective and patient-centric treatment strategies, underscore the significance of our work in advancing the understanding and care of fibromyalgia. Ultimately, this work contributes to the evolving field of personalized medicine, offering data-driven insights and evidence-based practices that can transform patient care.

## Figures and Tables

**Table 1:** Intra-algorithm and inter-algorithm adjusted Rand index (ARI) scores using 10 random seeds.

Intra-Algorithm adj. Rand Index	k = 2	k = 3	K = 4	K = 5	Intra-Algorithm adj. Rand Index	k = 2	k = 3	k = 4	k = 5
K-means	0.998	0.977	0.893	0.722	K-means, Gaussian	0.751	0.615	0.590	0.574
Gaussian	0.990	0.747	0.673	0.597	K-means Gower, Gaussian Gower	0.764	0.743	0.931	0.900
K-means Gower	1	0.999	1	0.985	K-means Gower, Gaussian	0.756	0.537	0.561	0.525
Gaussian Gower	1	0.739	0882	0.80	K-means, Gaussian Gower	0.751	0.674	0.6239	0.543
Gaussian X Gaussian Gower	0.978	0.609	0.558	0.518	AggWard, K-means	0.764	0.743	0.93	0.9007
K-means X K-means Gower	0.944	0.819	0.642	0.546	AggWard, gaussian	0.520	0.367	0.33	0.297
AggWard	1	1	1	1	AggComplete, K-means	−0.0001	−0.0002	0.0013	0.0068
AggComplete	1	1	1	1	Agg Complete, Gaussian	0.00005	0.0005	0.0045	0.0106
AggAverage	1	1	1	1	Agg Average, K-means	−0.0001	0.0020	0.0008	0.0014
AggComplete Gower	1	1	1	1	Agg Average, Gaussian	0.00005	0.0018	0.0025	0.0018
AggAvg Gower	1	1	1	1	Agg Single, K-means	−0.0001	−0.0002	0.0005	0.0013

## Data Availability

Data are contained within the article and Appendix A.

## Appendix A. Supplementary Analyses

To further investigate the impact of the choice of linkage criterion on the results of agglomerative clustering, we present in Figure A2 a dendrogram of the hierarchical clustering algorithm using different linkage criteria. As can be observed from the dendrogram, the choice of linkage criterion has a significant impact on the results of agglomerative clustering. Different linkage criteria can produce different cluster assignments for the same data set. This reinforces our previous conclusion that using linkage criteria other than Ward’s may result in less meaningful cluster assignments and highlights the importance of carefully selecting an appropriate linkage criterion when using agglomerative clustering.

Figure A3, Figure A4 and Figure A5 present the scores of the different algorithms, for the different values of k, for each of the SI, CHI, and DBI scores respectively.

Figure A6, Figure A7 and Figure A8: Distribution of SI, CHI, and DBI scores for K-means and Gaussian mixture with and without Gower’s distance metric for the different values of k, sampling 100% of the data, when using different starting seeds.

For the SI score, Figure A6 shows that using the Gower distance metric in K-means clustering improved performance for all values of k (as was shown in Semantic Assessment of Clustering Methods using 70% of the Data) and reduced the variance in performance across seeds. For Gaussian mixture, using Gower’s distance metric reduced variance only for k = 2.

For the CHI score Figure A7 indicates that using the Gower distance metric in K-means clustering decreased the average performance for all values of k but only reduced the variance in performance for k = 2 and k = 5.

For the BDI score, Figure A8 shows that using the Gower distance metric increased variance and produced worse results.

**Table A1:** Number of bad clustering defined as clusters containing less than 5% of data by each algorithm for each k, when using random subsets of 70% of the data.

Bad Clusterings	K = 2	K = 3	K = 4	K = 5
K-means	0	0	0	18
K-means Gower	0	0	0	5
Gaussian	2	3	13	16
Gaussian Gower	0	0	1	4
Agglomerative Ward	0	5	16	34
Agglomerative average	30	60	90	120
Agglomerative average Gower	30	60	90	120
Agglomerative single	30	60	90	120
Agglomerative single Gower	30	60	90	120
Agglomerative complete	29	51	80	109
Agglomerative complete Gower	1	16	28	47

**Table A2:** Pearson’s chi-square (2) test for k = 2 was used to determine whether or not the distributions of the two clusters differed significantly within each categorical feature.

Features	Variable	Cluster_0	Cluster_1	*p* Values
Sex	Yes	656 (89.74)	587 (91.86)	0.1766
	No	75 (10.26)	52 (8.14)	
Other rheumatic dis.	Yes	123 (16.83)	63 (9.86)	<0.001
	No	608 (83.17)	576 (90.14)	
Behcet’s disease	Yes	15 (2.05)	3 (0.47)	0.0103
	No	716 (97.95)	636 (99.53)	
FMF	Yes	25 (3.42)	16 (2.5)	0.3208
	No	706 (96.58)	623 (97.5)	
Inflammatory muscle disease	Yes	25 (3.42)	7 (1.1)	0.0045
	No	706 (96.58)	632 (98.9)	
Vasculitis	Yes	4 (0.55)	7 (1.1)	0.2566
	No	727 (99.45)	632 (98.9)	
Osteoarthritis	Yes	36 (4.92)	27 (4.23)	0.5375
	No	695 (95.08)	612 (95.77)	
Scleroderma	Yes	1 (0.14)	2 (0.31)	0.4864
	No	730 (99.86)	637 (99.69)	
Pseudogout	Yes	1 (0.14)	0 (0.0)	0.3496
	No	730 (99.86)	639 (100.0)	
Gout	Yes	6 (0.82)	7 (1.1)	0.6009
	No	725 (99.18)	632 (98.9)	
SLE	Yes	22 (3.01)	20 (3.13)	0.8975
	No	709 (96.99)	619 (96.87)	
Sjogren’s	Yes	12 (1.64)	11 (1.72)	0.9086
	No	719 (98.36)	628 (98.28)	
Arthritis related to IBD	Yes	11 (1.5)	9 (1.41)	0.8821
	No	720 (98.5)	630 (98.59)	
Spondyloarthritis	Yes	7 (0.96)	4 (0.63)	0.4926
	No	724 (99.04)	635 (99.37)	
Ankylosing spondylitis	Yes	10 (1.37)	11 (1.72)	0.5953
	No	721 (98.63)	628 (98.28)	
Psoriatic arthritis	Yes	36 (4.92)	20 (3.13)	0.0942
	No	695 (95.08)	619 (96.87)	
RA	Yes	58 (7.93)	44 (6.89)	0.4608
	No	673 (92.07)	595 (93.11)	
No other rheumatological condition	Yes	443 (60.6)	429 (67.14)	0.0121
	No	288 (39.4)	210 (32.86)	
Endometriosis	Yes	50 (6.84)	20 (3.13)	0.0019
	No	681 (93.16)	619 (96.87)	
Chronic sinusitis	Yes	66 (9.03)	47 (7.36)	0.2613
	No	665 (90.97)	592 (92.64)	
Asthma	Yes	78 (10.67)	43 (6.73)	0.0103
	No	653 (89.33)	596 (93.27)	
Allergy	Yes	173 (23.67)	108 (16.9)	0.002
	No	558 (76.33)	531 (83.1)	
Liver disease	Yes	16 (2.19)	15 (2.35)	0.8439
	No	715 (97.81)	624 (97.65)	
Obesity	Yes	181 (24.76)	140 (21.91)	0.2139
	No	550 (75.24)	499 (78.09)	
Uveitis	Yes	41 (5.61)	22 (3.44)	0.0562
	No	690 (94.39)	617 (96.56)	
Anemia	Yes	114 (15.6)	73 (11.42)	0.0249
	No	617 (84.4)	566 (88.58)	
Obstructive sleep apnea	Yes	62 (8.48)	29 (4.54)	0.0035
	No	669 (91.52)	610 (95.46)	
Renal disease	Yes	9 (1.23)	8 (1.25)	0.9724
	No	722 (98.77)	631 (98.75)	
Chronic headache	Yes	283 (38.71)	163 (25.51)	<0.001
	No	448 (61.29)	476 (74.49)	
Psychiatric conditions	Yes	171 (23.39)	100 (15.65)	<0.001
	No	560 (76.61)	539 (84.35)	
Endocrinological conditions	Yes	55 (7.52)	35 (5.48)	0.1272
	No	676 (92.48)	604 (94.52)	
Hyperthyroidism	Yes	21 (2.87)	11 (1.72)	0.1593
	No	710 (97.13)	628 (98.28)	
Hypothyroidism	Yes	70 (9.58)	83 (12.99)	0.0454
	No	661 (90.42)	556 (87.01)	
Dermatological conditions	Yes	68 (9.3)	45 (7.04)	0.1293
	No	663 (90.7)	594 (92.96)	
Malignancy	Yes	7 (0.96)	7 (1.1)	0.8002
	No	724 (99.04)	632 (98.9)	
Irritable bowel syndrome	Yes	229 (31.33)	163 (25.51)	0.0174
	No	502 (68.67)	476 (74.49)	
Inflammatory bowel disease	Yes	37 (5.06)	25 (3.91)	0.3073
	No	694 (94.94)	614 (96.09)	
Peptic ulcers	Yes	69 (9.44)	29 (4.54)	<0.001
	No	662 (90.56)	610 (95.46)	
Pulmonary disease	Yes	29 (3.97)	10 (1.56)	0.0076
	No	702 (96.03)	629 (98.44)	
Hyperlipidemia	Yes	94 (12.86)	85 (13.3)	0.8083
	No	637 (87.14)	554 (86.7)	
Hypertension	Yes	91 (12.45)	77 (12.05)	0.8224
	No	640 (87.55)	562 (87.95)	
Cardiovascular diseases	Yes	25 (3.42)	10 (1.56)	0.0299
	No	706 (96.58)	629 (98.44)	
Diabetes	Yes	60 (8.21)	34 (5.32)	0.035
	No	671 (91.79)	605 (94.68)	
Having a steady job	Yes	378 (51.71)	419 (65.57)	<0.001
	No	353 (48.29)	220 (34.43)	
Fibromyalgia had worsened in the past year	Yes	633 (86.59)	468 (73.24)	<0.001
	No	98 (13.41)	171 (26.76)	
Emotional trauma before fibromyalgia onset	Yes	428 (58.55)	373 (58.37)	0.947
	No	303 (41.45)	266 (41.63)	
Physical trauma before fibromyalgia onset	Yes	279 (38.17)	201 (31.46)	0.0094
	No	452 (61.83)	438 (68.54)	
Emotional trauma after fibromyalgia onset	Yes	383 (52.39)	282 (44.13)	0.0023
	No	348 (47.61)	357 (55.87)	
Physical trauma after fibromyalgia onset	Yes	162 (22.16)	81 (12.68)	<0.001
	No	569 (77.84)	558 (87.32)	
Pain causing awakening from sleep	Yes	651 (89.06)	479 (74.96)	<0.001
	No	80 (10.94)	160 (25.04)	
Waking up tired	Yes	713 (97.54)	605 (94.68)	0.0057
	No	18 (2.46)	34 (5.32)	
Other sleep problems	Yes	90 (12.31)	40 (6.26)	<0.001
	No	641 (87.69)	599 (93.74)	
Sleeping medication	Yes	201 (27.5)	142 (22.22)	0.0246
	No	530 (72.5)	497 (77.78)	
Bad dreams	Yes	208 (28.45)	105 (16.43)	<0.001
	No	523 (71.55)	534 (83.57)	
Snoring/coughing	Yes	157 (21.48)	81 (12.68)	<0.001
	No	574 (78.52)	558 (87.32)	
Waking up in the middle of the night/early morning	Yes	566 (77.43)	413 (64.63)	<0.001
	No	165 (22.57)	226 (35.37)	
Inability to breathe comfortably	Yes	137 (18.74)	61 (9.55)	<0.001
	No	594 (81.26)	578 (90.45)	
Grinding/clenching teeth	Yes	226 (30.92)	180 (28.17)	0.2666
	No	505 (69.08)	459 (71.83)	
Problems maintaining sleep	Yes	596 (81.53)	417 (65.26)	<0.001
	No	135 (18.47)	222 (34.74)	
Problems falling asleep	Yes	470 (64.3)	289 (45.23)	<0.001
	No	261 (35.7)	350 (54.77)	
Left foot	Yes	721 (98.63)	340 (53.21)	<0.001
	No	10 (1.37)	299 (46.79)	
Right foot	Yes	720 (98.5)	335 (52.43)	<0.001
	No	11 (1.5)	304 (47.57)	
Lower leg: left	Yes	706 (96.58)	173 (27.07)	<0.001
	No	25 (3.42)	466 (72.93)	
Lower leg: right	Yes	701 (95.9)	169 (26.45)	<0.001
	No	30 (4.1)	470 (73.55)	
Upper leg: left	Yes	709 (96.99)	244 (38.18)	<0.001
	No	22 (3.01)	395 (61.82)	
Upper leg: right	Yes	698 (95.49)	253 (39.59)	<0.001
	No	33 (4.51)	386 (60.41)	
Left buttock	Yes	688 (94.12)	165 (25.82)	<0.001
	No	43 (5.88)	474 (74.18)	
Right buttock	Yes	696 (95.21)	160 (25.04)	<0.001
	No	35 (4.79)	479 (74.96)	
Pelvis	Yes	716 (97.95)	330 (51.64)	<0.001
	No	15 (2.05)	309 (48.36)	
Lower back	Yes	726 (99.32)	474 (74.18)	<0.001
	No	5 (0.68)	165 (25.82)	
Upper back	Yes	714 (97.67)	341 (53.36)	<0.001
	No	17 (2.33)	298 (46.64)	
Abdomen	Yes	689 (94.25)	204 (31.92)	<0.001
	No	42 (5.75)	435 (68.08)	
Chest	Yes	684 (93.57)	122 (19.09)	<0.001
	No	47 (6.43)	517 (80.91)	
Left wrist	Yes	722 (98.77)	303 (47.42)	<0.001
	No	9 (1.23)	336 (52.58)	
Right wrist	Yes	722 (98.77)	354 (55.4)	<0.001
	No	9 (1.23)	285 (44.6)	
Lower arm: left	Yes	718 (98.22)	208 (32.55)	<0.001
	No	13 (1.78)	431 (67.45)	
Lower arm: right	Yes	713 (97.54)	220 (34.43)	<0.001
	No	18 (2.46)	419 (65.57)	
Upper arm: left	Yes	697 (95.35)	102 (15.96)	<0.001
	No	34 (4.65)	537 (84.04)	
Upper arm: right	Yes	695 (95.08)	113 (17.68)	<0.001
	No	36 (4.92)	526 (82.32)	
Left shoulder	Yes	722 (98.77)	334 (52.27)	<0.001
	No	9 (1.23)	305 (47.73)	
Right shoulder	Yes	716 (97.95)	327 (51.17)	<0.001
	No	15 (2.05)	312 (48.83)	
Left jaw	Yes	678 (92.75)	141 (22.07)	<0.001
	No	53 (7.25)	498 (77.93)	
Right jaw	Yes	683 (93.43)	144 (22.54)	<0.001
	No	48 (6.57)	495 (77.46)	
Left head	Yes	695 (95.08)	163 (25.51)	<0.001
	No	36 (4.92)	476 (74.49)	
Right head	Yes	699 (95.62)	161 (25.2)	<0.001
	No	32 (4.38)	478 (74.8)	
Neck	Yes	727 (99.45)	446 (69.8)	<0.001
	No	4 (0.55)	193 (30.2)	
All body	Yes	629 (86.05)	0 (0.0)	<0.001
	No	102 (13.95)	639 (100.0)	
Dizziness	Yes	501 (68.54)	280 (43.82)	<0.001
	No	230 (31.46)	359 (56.18)	
Nausea/vomiting	Yes	336 (45.96)	172 (26.92)	<0.001
	No	395 (54.04)	467 (73.08)	
Abdominal pain	Yes	467 (63.89)	301 (47.1)	<0.001
	No	264 (36.11)	338 (52.9)	
Constipation	Yes	334 (45.69)	200 (31.3)	<0.001
	No	397 (54.31)	439 (68.7)	
Heartburn	Yes	297 (40.63)	179 (28.01)	<0.001
	No	434 (59.37)	460 (71.99)	
Taste disorder	Yes	169 (23.12)	38 (5.95)	<0.001
	No	562 (76.88)	601 (94.05)	
Smell disorder	Yes	198 (27.09)	69 (10.8)	<0.001
	No	533 (72.91)	570 (89.2)	
Xerostomia	Yes	403 (55.13)	221 (34.59)	<0.001
	No	328 (44.87)	418 (65.41)	
Epistaxis	Yes	84 (11.49)	46 (7.2)	0.0068
	No	647 (88.51)	593 (92.8)	
Frequent urges to urinate	Yes	364 (49.79)	212 (33.18)	<0.001
	No	367 (50.21)	427 (66.82)	
Tinnitus	Yes	343 (46.92)	198 (30.99)	<0.001
